# Effectiveness of Web-Based Interventions on Clinical Outcomes and Lifestyle Modifications in Women Planning to Conceive: A Systematic Review

**DOI:** 10.3390/healthcare13091037

**Published:** 2025-05-01

**Authors:** Hitomi Suzuki, Phyu Phyu Tun, Shuxian Liu, Erika Ota, Naoko Arata

**Affiliations:** 1 Division of Women’s Internal Medicine, Integrated Center for Women’s Health, National Center for Child Health and Development, Tokyo 157-8535, Japan; arata-n@ncchd.go.jp; 2 Global Health Nursing, Graduate School of Nursing, St. Luke’s International University, Tokyo 104-0044, Japan; 22dn901@slcn.ac.jp (P.P.T.); 24dn020@slcn.ac.jp (S.L.); ota@slcn.ac.jp (E.O.); 3 Tokyo Foundation for Policy Research, Tokyo 106-0032, Japan

**Keywords:** preconception care, primary care, public health, behavior change, lifestyle intervention

## Abstract

**Purpose:** to identify evidence on the effectiveness of web-based interventions for lifestyle modification among women or couples of reproductive ages wishing to conceive. **Methods:** A systematic search was conducted in February 2023 across CENTRAL, PubMed, Web of Science, Embase, Emcare, ClinicalTrials.gov, and WHO ICTRP. Data from four randomized controlled trials involving 1965 preconception women were narratively synthesized following risk of bias assessment. Interventions included a web-based conversational agent system, an email-based mobile service, and a mobile app providing lifestyle-related information. **Results:** Despite diverse assessment tools, benefits were observed for systolic blood pressure, serum folate levels, and physical activity. However, no significant effects were found for intake of vegetables and fruit, folic acid supplementation, smoking, alcohol consumption, waist circumference, weight, BMI, overweight status, HbA1c, total cholesterol, HDL, stress, depression, anxiety, or pregnancy outcomes. **Conclusions:** Web-based interventions show potential in improving certain health behaviors among preconception women. Further high-quality studies are needed to assess their effectiveness on a broader range of outcomes, including dietary habits, physical activity, and substance use, and to inform their integration into preconception care strategies. **Registration:** We registered the study protocol with PROSPERO (CRD42023488277).

## 1. Introduction

“Preconception health” refers to the overall health status of non-pregnant individuals of reproductive age. The importance of preconception care is highlighted by its potential to improve health conditions, including the next generation’s health. It involves comprehensive interventions that address biological, behavioral, and social aspects [1,2]. Emerging evidence of a strong association between preconception health and maternal and child health outcomes has shown that maternal nutrition and health status are a pivotal determinant of embryonic and fetal development. Maternal eating habits and lifestyle have been demonstrated to have a significant impact on short- and long-term health outcomes for both mother and child [3]. Unhealthy dietary intake and inappropriate lifestyle habits can contribute to future lifestyle-related diseases in people of reproductive age, as well as affect fertility and pregnancy outcomes [4]. The preconception period is seen as a unique opportunity not only to improve their own health but to reduce risk factors associated with non-communicable diseases for the next generations, as represented by the Developmental Origins of Health and Disease paradigm [5,6]. Preconception care includes consultations on lifestyle choices, such as family planning and contraceptive provision, lifestyle modification including nutrition, immunization, infection control, and chronic disease control, and the use of substances such as tobacco and alcohol; such consultations are necessary throughout life for most people. Lifestyle habits such as food intake, folic acid intake, physical activity, and stress management are elements of preconception care that can be self-managed. In addition, a study in the UK reported that nine out of ten women of reproductive age had at least one risk factor [7], pointing to the need for preconception care as primary care [8] and indicating it should be more accessible to preconception youth, who would benefit from it [9].

The traditional health care model that Baby Boomers (born 1955–1964) and Generation X (born 1965–1980) were accustomed to is not particularly attractive to Millennials and Generation Z (born after 1980s), who, with on-demand access to health information, prefer a more flexible approach to health care [10]. Since 2000, young people have exhibited a recurring trend of moving in and out of urban neighborhoods [11]. The increasingly favored semi-nomadic lifestyle among the younger generation, with short periods of displacement, demands a more convenient experience, with full autonomy over their health. A study conducted in Brazil found that low-income young people who participated in the survey spent more time on the Internet than higher-income young people, as their socio-economic status did not prevent or limit their access to the Internet. It suggests that access to the Internet is no longer a barrier in low-income families, and new communication technologies play a central role in the lives of most “digital natives” and in the search for health information [10].

Given this context, it is assumed that strategies utilizing web-based resources, including mobile applications, may be effective for a public health approach to comprehensive health care targeting the young preconception generation. Musgrave et al. conducted a systematic review to address preconception behavior change through mobile phone apps [12]. Due to the limited number of studies and low certainty of evidence, the effectiveness of mobile phone app interventions in promoting positive behavior changes among women of reproductive age prior to conception is inconclusive. Despite the growing popularity of mobile apps, web-based services are also accessible via mobile phones and offer preconception care. The previous review covered both preconception and interconception periods, but significant differences may exist between these periods, necessitating a focus on the preconception period.

Therefore, this research aimed to identify evidence for the effectiveness of web-based interventions, including mobile apps, aimed at lifestyle modification for women or couples of reproductive ages who wish to conceive.

## 2. Methods

### 2.1. Study Design

This systematic review was performed in accordance with the *Cochrane Handbook* [13] and followed the Preferred Reporting Items for Systematic Reviews and Meta-Analyses (PRISMA) 2020 statement for methods of reporting. The review protocol was registered on the international prospective register of systematic reviews, PROSPERO (https://www.crd.york.ac.uk/prospero/ (accessed on 27 April 2025)) (CRD42023488277). The research question was set as “What is the effectiveness of web-based interventions in improving clinical outcomes and lifestyle factors for women who want to conceive?”.

### 2.2. Eligibility Criteria

We included individual and cluster randomized controlled trials (RCTs) involving reproductive-aged (15–49 years old) women or couples who wish to conceive, including infertile women or couples, infertility treatment, artificial reproductive technologies (ARTs), and obesity, but excluded chronic diseases. Although the inclusion of women with subfertility or undergoing ART may introduce heterogeneity, these populations represent a key subgroup actively seeking conception and are likely to benefit from lifestyle interventions. Including them enhances the clinical relevance and generalizability of the review findings by reflecting the diversity of individuals in the preconception period. We included web-based interventions and preconception care services, including mobile phone-based services, and compared them with standard care or no intervention. Preconception care also includes health guidance, consultation, advice, health promotion education, health checkups, coaching through mobile health (mHealth), Internet of things (IoT), computer-delivered intervention, text messages, and mobile apps. The exclusion criteria featured studies published in languages other than English, research designs that were not RCTs, and studies without any available full text from the included databases or the original author.

### 2.3. Literature Search

In February 2023, a search was conducted in the Cochrane Central Register of Controlled Trials (CENTRAL) (The Cochrane Library), PubMed (include MEDLINE), Web of Science, Embase, Emcare, ClinicalTrial.gov, and WHO ICTRP with no date/time specified. The search strategy is provided in Supplementary File S1. The language was restricted to only English. We conducted a manual search for relevant papers and searched the reference lists of all included studies. The construction of the search strategy was informed by the PICO framework, as presented in Table 1.

### 2.4. Study Identification and Data Extraction

Two reviewers (H.S. and P.P.T.) independently screened all potential studies in two stages, abstract and full-text screening, using Rayyan for Systematic Reviews (https://www.rayyan.ai/ (accessed on 27 April 2025)). We resolved discrepancies through discussion. Data extraction was also conducted independently by two reviewers (H.S. and P.P.T.) who consulted the third reviewer (E.O.) if discrepancies were found. For each eligible review, the following items were extracted: study settings, study design, research population, number of participants, intervention methods, control methods, and outcomes.

### 2.5. Risk of Bias Assessment

In this study, only RCTs were used as selection criteria and the Cochrane Risk of Bias tool 2.0 was used to assess the risk of bias [14]. Two reviewers evaluated the potential for bias in each study independently, using appropriate evaluation methods.

### 2.6. Data Synthesis

As the studies included in this review reported different outcomes, with no two studies reporting the same outcomes, it was not possible to conduct a meta-analysis. Therefore, we performed a descriptive analysis and presented the findings narratively. In this review, studies were classified and analyzed according to the primary and secondary outcome measurements.

### 2.7. Ethical Approval

As this is a systematic review of published papers or data, no ethical approval is required to conduct this review.

## 3. Results

### 3.1. Study Selection

**Figure 1:**
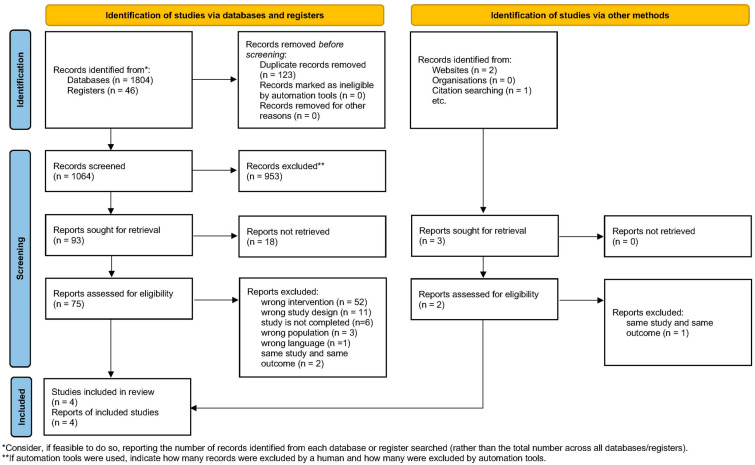
PRISMA flow diagram of selection study.

From 1850 identified records, 123 duplicates were removed, and 953 articles were excluded based on title and abstract screening. We excluded 52 studies because of wrong interventions, 11 studies because of wrong study designs, 6 because the studies are not completed, 3 because of wrong population, 1 because of wrong language, and 2 because of duplication. This left 93 articles to be assessed for eligibility. An additional hand search based on the relevant or cited articles identified three records. We excluded one full-text study because of duplication. Finally, a total of 78 available full-text studies were screened, and four articles were included in this systematic review. The flow diagram of the study selection process is shown in Figure 1. As recommended by PRISMA, the list of excluded full-text articles is presented in the Supplementary File S2 along with the reasons for exclusion.

### 3.2. Study Characteristics

A summary of the included studies is presented in Table 2. All of the included studies were individual randomized controlled trials (RCTs) [15,16,17,18]. The settings of the included studies were the USA [16], UK [17], Netherlands [18], and Malaysia [15]. The participants’ capacity varied from 262 to 626 women. One study used an online advertisement for recruiting [16], and three studies recruited from hospital settings or medical centers [15,17,18]. Two studies included women of broadly reproductive age [17,18], one study included women aged 18–34 years [16], and one included woman aged 20–39 [15]. All four studies included non-pregnant women [15,16,17,18], and two studies included women undergoing fertility treatment [17,18]. Outcomes were assessed between 12 and 48 weeks after randomization, baseline, or intervention.

### 3.3. Intervention Characteristics

In terms of intervention formats, one study utilized a web-based conversational agent for intervention [16], while the other three studies were implemented through smartphone apps [15,17,18]. Among these, two studies provided participants with personalized lifestyle guidance and information via the app, displaying their current lifestyle status to motivate adherence [17,18]. Another study offered young couples optional lifestyle challenges and information on healthy lifestyles through a smartphone app [15].

Regarding the intervention content, one study focused on 112 risks categorized into 13 domains (emotional and mental health, environmental issues, genetic health history, health care and programs, health conditions and medicines, immunizations and vaccines, infectious diseases, men and health care, nutrition and activity, relationships, reproductive health, substance use, and family planning) [16]. Another study concentrated more on dietary and physical activity behaviors [15]. The remaining two studies focused on addressing inadequate intake of vegetables and fruits, lack of folic acid supplementation, and unhealthy alcohol and smoking habits [17,18].

Regarding intervention participation and adherence, Hanafiah et al., 2022 [15] did not report adherence or participants’ usage of the app. The follow-up completion rate was 70.0% in the intervention group and 74.0% in the control group. Jack et al., 2020 [16] conducted the intervention using the Gabby system and reported that after 12 months, 199 out of 262 women in the intervention group (76%) had interacted with Gabby at least once. The median number of logins was six (IQR 8). The average duration of a single session was 14.5 min (SD 6.3). The total average interaction time for each woman using Gabby (excluding risk assessments) was 123.5 min (SD 155.0). At 6 months, 76 (64%) of 118 respondents rated Gabby as easy to use, 75 (68%) of 110 respondents trusted Gabby (much or very much), and 94 (81%) of 116 respondents indicated that Gabby answered their questions. The follow-up completion rate was 88.5% in the intervention group and 86.8% in the control group. Ng et al., 2021 [17] did not report adherence or participants’ usage of the app. The follow-up completion rate was 70.2% in the intervention group and 73.3% in the control group. Oostingh et al., 2020 [18] reported that of the 626 randomized women, 468 completed the program, resulting in an overall compliance rate of 74.8%: 211 in the intervention group (68.5%) and 257 in the control group (80.8%; *p* < 0.001). Among the 222 randomized men, 176 completed the program, with an overall compliance rate of 79.3%: 78 in the intervention group (73.6%) and 98 in the control group (84.5%; *p* = 0.045). The follow-up completion rate was 96.9% in the intervention group and 96.8% in the control group.

Of the included studies, one used a web-based conversational agent system for preconception care to reduce preconception health risks [16]. In this previous study, 112 risks were categorized into 13 domains (emotional and mental health, environmental issues, genetic health history, health care and programs, health conditions and medicines, immunizations and vaccines, infectious diseases, men and health care, nutrition and activity, relationships, reproductive health, substance use, and family planning). Screening was conducted and interventions were provided to modify selected risk behaviors [16]. In two studies, lifestyle modifications for subfertile women focused on addressing inadequate intake of vegetables and fruits, lack of folic acid supplementation, and undesirable alcohol and smoking habits, and email-based mobile services were used [17,18]. The final study included was a mobile app providing information on lifestyle challenges and healthy lifestyles with a focus on healthy foods and physical activity [15].

To enable comparisons between studies, the outcomes from the main intervention approaches reported in the included studies were categorized as follows: (a). food intake (vegetable and fruit intake) [15,16,17,18]; (b). folic acid intake [16,17,18]; (c). smoking and alcohol consumption [16,17,18]; (d). physical activity [15,16]; (e). stress management [15,16].

Hanafiah et al. (2022) [15] used the amount consumed during the week for vegetable and fruit intake and the total number of minutes exercised during the week for exercise. This study also reported on stress using Depression Anxiety and Stress Scale 21-item (DASS-21) scores, calculating the number of people rated as moderate or severe [19]. Jack et al. (2020) [16] assessed the five stages of change in the Prochaska’s transtheoretical model by score [20]. Ng et al. (2021) [17] evaluated the Rotterdam Reproduction Risk Score (R3-score) and its conversion to a risk score developed based on Preconception Dietary Risk score (PDR) [21]. Lower scores corresponded to a healthier lifestyle. Fruit and vegetable intake was subdivided into risk scores ranging from “0” (adequate daily intake, at least two fruits and 200 g vegetables per day) to “3” (inadequate daily intake, less than 1.5 fruits and 150 g of vegetables per day). Folic acid intake was defined as “0” (adequate, 400 µg of folic acid recommended for the perinatal period of pregnancy) or “3” (inadequate). Smoking was subdivided into scores from “0” (no smoking) to “6” (15 or more cigarettes per day) and alcohol intake from “0” (no alcohol intake) to “3” (three or more alcoholic beverages per day). Oostingh et al. (2020) [18] reported the number of people with events above a threshold.

### 3.4. Quality Assessment of the Included Studies

**Figure 2:**
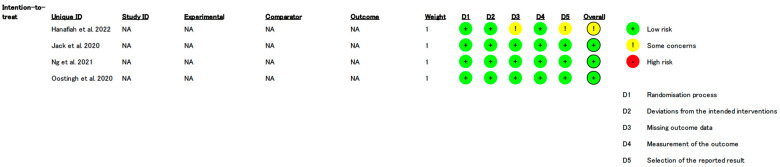
Risk of bias assessment [15,16,17,18].

The detailed risk of bias assessment based on the Risk of Bias 2.0 (RoB 2.0) tool is presented in Figure 2. All four studies [15,16,17,18] were judged to have a low risk of bias or some concerns in the following domains: Domain 1: Bias arising from randomization process. All four studies were low risk because the generation and allocation of randomization sequences were appropriately reported. In Domain 2: regarding bias due to deviation from the intended intervention, all studies considered that, although blinding of participants and researchers was not possible due to the nature of the intervention, there was no deviation due to allocation [15,16,17,18]. Regarding Domain 3: Bias due to missing outcome data. One study [15] was rated as having some concern for bias due to high dropout rates of 46.7% in the intervention group and 42.0% in the control group. These dropout rates may have affected the reliability of the results. The remaining studies were rated as low risk for bias in this domain [16,17,18]. For Domain 4: Bias in measurement of the outcome. All studies were rated as having low risk of bias, as outcome assessors, including statisticians and researchers, were adequately blinded [15,16,17,18]. In Domain 5: Bias in selection of the reported result. All studies were judged to be at low risk of bias because reported outcomes matched the registered protocols, and no evidence of selective reporting was identified [15,16,17,18]. Overall, three studies were assessed as having a low risk of overall bias [16,17,18]. On the other hand, one study [15] was assessed as having some concerns about the overall risk of bias due to the lack of important outcome data and the inability to blind participants and study staff.

### 3.5. Intervention Outcomes

The summary of reported outcomes is shown in Table 3, which consists of the clinical outcomes and lifestyle change outcomes. The clinical outcomes can be categorized into anthropometric indicators [15,16], physiological and biochemical indicators [15,18], mental health status [15,16], and pregnancy-related outcomes [17,18]. In contrast, the lifestyle change outcomes can be divided into food intake (vegetable and fruit intake) [15,16,17,18], folic acid intake [16,17,18], smoking and alcohol consumption [16,17,18], and physical activity [15,16].

#### 3.5.1. Anthropometric Indicators

Hanafiah et al. (2022) [15] reported no significant differences between the intervention group (IG) and the control group (CG) in waist circumference, weight, and BMI at 36 weeks. The mean (SD) waist circumference was 82.6 (13.9) cm in the IG and 81.7 (13.9) cm in the CG (*p* = 0.547), while the mean weight was 66.7 (18.8) kg in the IG compared to 65.4 (17.5) kg in the CG (*p* = 0.528). The mean BMI was 27.3 (7.4) in the IG and 27.0 (7.0) in the CG (*p* = 0.701). However, a significant difference was observed in systolic blood pressure, with the IG showing a higher mean (107.6 [14.0] mmHg) compared to the CG (104.7 [10.6] mmHg, *p* = 0.031), while diastolic blood pressure differences were not significant (*p* = 0.230). Jack et al. (2020) [16] reported missing data for the underweight group in the IG, rendering data unavailable. For overweight status, no significant differences were found at 24 weeks (IG mean = 3.85 vs. CG mean = 3.64, *p* = 0.30) or at 48 weeks (IG mean = 4.02 vs. CG mean = 4.05, *p* = 0.88). Ng et al. (2021) [17] and Oostingh et al. (2020) [18] did not report anthropometric outcomes.

#### 3.5.2. Physiological and Biochemical Indicators

Hanafiah et al. (2022) [15] assessed several biochemical markers at 36 weeks, finding no significant differences between groups. HbA1c levels were 5.3 (0.6) in the IG and 5.2 (0.5) in the CG (*p* = 0.093). Total cholesterol was 4.86 (0.9) mmol/L in the IG compared to 4.79 (0.9) mmol/L in the CG (*p* = 0.527), and HDL levels were 1.45 (0.4) mmol/L in the IG versus 1.43 (0.4) mmol/L in the CG (*p* = 0.632). Ng et al. (2021) [17] reported serum folate levels at 12 weeks, showing a higher median in the IG (48.6 nmol/L, IQR 28.8–64.1) compared to the CG (30.1 nmol/L, IQR 17.9–51.9). Jack et al. (2020) [16] and Oostingh et al. (2020) [18] did not report physiological or biochemical outcomes.

#### 3.5.3. Mental Health Status

Mental health outcomes were assessed by Hanafiah et al. (2022) [15] using the DASS-21 scale at 36 weeks. Stress levels showed no significant differences, with moderate stress reported in 15.9% of the IG and 16.3% of the CG and severe stress in 2.1% of the IG versus 6.3% of the CG (*p* = 0.19). Moderate depression was observed in 34.5% of the IG and 33.1% of the CG, while severe depression was reported in 2% of the IG and 6% of the CG (*p* = 0.64). Anxiety levels were also similar between groups (moderate: IG 20.0% vs. CG 19.4%; severe: IG 1.4% vs. CG 3.1%, *p* = 0.60). Jack et al. (2020) [16] measured stress using the Perceived Stress Scale (PSS) and potential depression using the PHQ-2. At 24 weeks, the IG had a mean stress score of 4.04 compared to 3.60 in the CG (*p* = 0.21), and at 48 weeks, the scores were 4.09 (IG) and 3.89 (CG, *p* = 0.52). Potential depression scores were also not significantly different at 24 weeks (IG mean = 3 vs. CG mean = 2.77, *p* = 0.66) or 48 weeks (IG mean = 3.27 vs. CG mean = 3.58, *p* = 0.41). Mental health outcomes were not reported in Ng et al. (2021) [17] or Oostingh et al. (2020) [18].

#### 3.5.4. Pregnancy-Related Outcomes

Pregnancy-related outcomes were reported by Ng et al. (2021) [17] and Oostingh et al. (2020) [18]. Ng et al. (2021) [17] observed pregnancy outcomes at 24 weeks, with a difference between IG and CG of 2.83 (95% CI: 0.35 to 57.76). Oostingh et al. (2020) [18] assessed pregnancy rates at 52 weeks, reporting a difference of 0.807 (95% CI: 0.574 to 1.134) between the IG and CG. Hanafiah et al. (2022) [15] and Jack et al. (2020) [16] did not report pregnancy-related outcomes.

#### 3.5.5. Food Intake (Vegetable and Fruit Intake)

Hanafiah et al. (2022) [15] did not discuss vegetable intake in the main text; however, the reported data showed that at 36 weeks, the mean ± standard deviation (SD) of vegetable intake frequency was 9.4 ± 7.8 times per week in the intervention group, compared to 8.7 ± 6.1 times per week in the control group. This difference was not statistically significant (*p* = 0.458) [15]. Ng et al. (2021) [17] reported that the difference in the vegetable intake risk score between intervention and control groups for the baseline was −0.21 (95% CI −0.48 to 0.03) at 12 weeks and 0.00 (95% CI −0.30 to 0.27) at 24 weeks. These results indicated no statistically significant differences [17]. Oostingh et al. (2020) only reported on adequate nutrition and lifestyle promotion in the appendix material and explained in the text that both the intervention and control groups showed more appropriate behavior and reduced (i.e., improved) DRS after 24 weeks of coaching [18]. The proportion of participants consuming more than 200 g of vegetables per day was slightly higher in the intervention group compared to the control group at both 24 weeks (40% vs. 28%) and 36 weeks (33% vs. 21%) [18].

For fruit intake, Hanafiah et al. (2022) [15] reported that the mean ± SD for the intervention group was 5.8 ± 4.8 times per week, and the control group mean ± SD was 5.1 ± 4.2 times per week (*p* = 0.199). No significant differences were found [15]. Ng et al. (2021) [17] assessed fruit intake risk scores and reported a difference of −0.14 (95% CI: −0.60 to 0.07) at 12 weeks and −0.21 (95% CI: −0.50 to 0.66) at 24 weeks between the intervention and control groups. Again, no significant differences were observed [17]. In the study by Oostingh et al. (2020) [18], fruit intake was not specifically discussed in the main text. Any relevant data appeared only in the Supplementary Materials or were discussed in the context of overall dietary or behavioral improvements [16,18]. Oostingh et al. (2020) showed a slightly higher percentage of participants consuming two or more fruits per day at 24 weeks (67% vs. 44%) and at 36 weeks (68% vs. 57%) in the intervention group [18].

Jack et al. (2020) [16] did not mention this in the main text of the article, and the appendix included results regarding stage of change (SoC) score of bad diet or food choices (<5 daily servings of fruits and vegetables and/or regular intake of junk food). Despite 374 out of 528 participants (71%) being nutritionally deficient, there was no significant difference in the effect of the intervention on fruit and vegetable intake at 24 weeks and 48 weeks [16].

#### 3.5.6. Folic Acid Intake

Jack et al. (2020) [16] only reported on the effects in the appendix material, with no mention of folic acid intake in the text of the article. Of the 528 participants, 300 (57%) were not taking folic acid supplements. The effect of the intervention on folic acid supplement intake was not significantly different for both 24 and 48 weeks [16]. Ng et al. (2021) reported that the difference in folate intake risk scores between the intervention and control groups at baseline was −0.04 (95% CI −0.29 to 0.21) at 12 weeks and −0.16 (95% CI −0.42 to 0.09) at 24 weeks, which was not significantly different [17]. Similarly, Oostingh et al. (2020) only reported on the effects in the appendix material, mentioning that the proportion of those taking adequate supplementary folic acid intake was 97% in the intervention group and 99% in the control group at 24 weeks and 96% in the intervention group and 96% in the control group at 36 weeks [18].

#### 3.5.7. Smoking and Alcohol Consumption

In the study by Jack et al. (2020) [16], both smoking and alcohol consumption were only reported in the appendix material and were not mentioned within the main body of the article: of the 528 participants, 56 (11%) women reported current tobacco use, and 188 (36%) were consuming excessive alcohol. The effects of the intervention on smoking and alcohol consumption were not significantly different for both 24 and 48 weeks [16]. Ng et al. (2021) reported no significant differences in smoking between the baseline intervention and control groups nor in alcohol consumption risk scores at 12 and 24 weeks, respectively [17]. Regarding smoking and alcohol consumption in the Oostingh et al. (2020) [18] study, the proportion of non-smokers was 93% in the intervention group and 92% in the control group at 24 weeks and 80% in the intervention group and 91% in the control group at 36 weeks. The proportion of those not consuming alcohol was 78% in the intervention group and 73% in the control group at 24 weeks and 69% in the intervention group and 75% in the control group at 36 weeks [18].

#### 3.5.8. Physical Activity

Hanafiah et al. (2022) [15] reported that there were significant differences between the intervention and control groups in job-related physical activity. The study showed 259.9 min/week in the intervention group and 153.8 min/week in the control group for vigorous activity (*p* = 0.03) and 749.0 min/week in the intervention group and 550.0 min/week in the control group for moderate activity (*p* = 0.058) [15]. Regarding exercise in the study by Jack et al. (2020) [16], 248 (47%) out of 528 participants did not exercise enough. There were no significant differences in exercise between the intervention and control groups for both 24 and 48 weeks [16].

## 4. Discussion

This systematic review narratively analyzed the effectiveness of web-based interventions to improve the lifestyle of women who wanted to conceive. Although the studies included in this review varied in their evaluation tools and outcome measures—rendering meta-analysis unfeasible—four high-quality randomized controlled trials (RCTs) employing web-based tools (including mobile platforms) were identified. This systematic review identified some evidence that web-based interventions may lead to modest improvements in physical activity, systolic blood pressure, and serum folate levels among women in the preconception period. However, no significant effects were found for other important behavioral and clinical outcomes, including the intake of vegetables and fruits, folic acid supplementation, smoking, alcohol consumption, anthropometric measures, metabolic indicators, mental health status, and pregnancy-related outcomes. These findings should be interpreted with caution given the small number of included studies, substantial heterogeneity in intervention content and delivery, and the presence of moderate to high risk of bias across several methodological domains. Future research should prioritize rigorously designed randomized controlled trials with adequate sample sizes, standardized outcome measures, and improved reporting to strengthen the evidence base for digital approaches to preconception care.

Regarding study quality, all included trials were rated as having a high risk of bias due to the lack of blinding of participants. This is a well-recognized challenge in behavioral intervention research, where the nature of the intervention often makes double-blinding impractical or impossible [22,23]. In the absence of blinding, participants may consciously or unconsciously report socially desirable behaviors or exaggerate improvements, particularly when outcomes are self-reported [24]. Such biases can compromise the internal validity of the findings and lead to overestimation of intervention effects. Therefore, these results should be interpreted with caution. To address this issue, future studies should consider employing strategies to minimize bias, such as blinding outcome assessors, limiting the disclosure of study hypotheses to participants, or incorporating standardized, automated intervention delivery methods where feasible [25,26]. In addition, one of the included studies reported a dropout rate exceeding 40%. Although the findings of this study suggested potentially significant effects on reducing systolic blood pressure and increasing vigorous job-related physical activity, the interpretation of these results should be approached with caution, as the high level of attrition may have introduced attrition bias and affected the reliability of these outcome estimates.

Although we considered using the GRADE approach to assess the certainty of the evidence, we ultimately decided not to present a formal Summary of Findings table. This decision was made since each outcome in this review was reported by only a single study, all with relatively small sample sizes. According to the GRADE framework, this leads to serious imprecision across all outcomes, resulting in overall evidence certainty rated as “low” or “very low”. Given the absence of pooled estimates or multiple studies per outcome, and the consistent downgrading due to imprecision, we judged that a formal GRADE table would not provide meaningful additional value to readers. Instead, we have provided detailed narrative assessments of study quality and evidence limitations throughout the Results and Discussion sections.

This systematic review included interventions developed to reduce preconception health risks through web-based conversational agent systems, email-based mobile services, and mobile apps. One study was selected from lifestyle habits as well as emotional and mental health, environmental issues, genetic health history, health care and programs, health conditions and medicines, immunizations and vaccines, infectious diseases, men and health care, nutrition and activity, relationships, reproductive health, substance use, and family planning and broader health risks, and it was uncertain whether interventions were necessarily lifestyle related [16]. There was no clear difference in overall lifestyle improvement between the intervention group, which used a web-based tool for lifestyle improvement, and the control group, with some outcomes showing effects. However, all reports used different outcomes, making it impossible to conduct a meta-analysis, and no clear evidence was obtained.

The 2018 Lancet series on preconception health highlights that the preconception period is a critical time in shaping pregnancy outcomes at conception as well as the health of the next generation of offspring [3,27,28]. Since new evidence on the impact of preconception health conditions was confirmed, there has been an increased focus on preconception health, and more studies related to preconception health are being developed worldwide. The results of the studies addressed in the present review did not report any apparent improvements in food intake or folic acid intake. Previous studies have noted that although women generally accept interventions regarding food intake and supplementation, intake is often hampered by poor adherence [28,29]. Studies on gestational diabetes mellitus (GDM) have found that adherence to a healthy dietary pattern pre-pregnancy is associated with a definite and significant reduction in GDM risk [30]. Practical strategies for ensuring adherence and improving food intake and supplementation behavior need to be explored.

In terms of physical activity, only one RCT reported significant differences between the intervention and control groups [15]. The World Health Organization recommends that for the average adult aged 18–64 years, 150–300 min per week of moderate–vigorous aerobic activity or 75–150 min of vigorous-intensity aerobic activity plus two days per week of strengthening muscle and bone [31] would be beneficial. Although, based on the rates reported in the included RCT within the range recommended by the WHO, there were significant differences in work-related vigorous physical activity and no significant differences in leisure time physical activity. This raises a question as to whether this increase in physical activity is associated with a positive outcome.

Most studies also point out that less than 50% of women adhere to physical activity prescription protocols [32,33]. Jack et al. (2020) also reported that the proportion of women not exercising was 47%, almost half [16]. Notably, preconception physical activity patterns were shown to determine exercise habits during pregnancy [34].

In this present review, there were no clear differences found between the intervention and control groups in terms of smoking and alcohol consumption. In this regard, Oostingh et al. (2020) stated that given the widely recognized negative effects of smoking and alcohol consumption on fertility and reproductive outcomes, infertile couples who are aiming to become pregnant and stop smoking and drinking are expected to have already done so [18]. Although Jack et al. (2020) reported that rates of smoking and excessive alcohol consumption are less frequent than other preconception health risks [16], the impact of smoking and alcohol consumption on pregnancy outcomes is considered strong, and there is a need to ensure risk reduction [35,36].

With regard to stress management, several previous studies have reported that increased levels of perceived stress and depressive symptoms are associated with difficulty conceiving and time to pregnancy taking longer [37,38]. Although the results of this review did not show the effect of interventions on stress management, they indicate that improving mental health may have a positive outcome on pregnancy outcomes, suggesting that further research is needed.

Barker et al. (2018) presented a conceptual model of the pathways from preconception to maternal and next-generation early childhood health that should be quantified when higher-quality data from randomized trials become available [28]. As preconception care aims to reduce behaviors and personal and environmental factors that contribute to poor maternal and child health, its ultimate purpose is to improve the health of mothers and their future children in the short and long term [2]. Although the lifestyle interventions considered in this review focused on short-term lifestyle improvements in preconception women and couples, the real outcomes are the long-term health of the subject’s own future, improving pregnancy and birth outcomes, and the health of the future generations. Evidence from related health domains, such as the high prevalence of urinary incontinence among physically active young women, further underscores the broader relevance of addressing lifestyle factors in women at reproductive age [39,40].

However, assessing the impact of interventions on long-term outcomes involves many other factors besides lifestyle, such as genetic history, infectious diseases and chronic diseases. There is a need to ensure that the evidence at each stage is clear, and this study will contribute to this.

### Limitations of the Study

The present review had several limitations. First, a major limitation of this review is the small number of included studies, which limits the generalizability of the findings. In addition, research design, heterogeneity of intervention components, and different outcome assessment tools across studies limit the feasibility of meta-analysis and reduce the reliability of the overall conclusions. Further quantitative studies are desirable for accumulating findings with a unified outcome assessment tool. Future research should aim to use standardized and validated outcome measures and adopt more consistent intervention protocols to improve comparability and facilitate quantitative synthesis. Second, the targeted study design does not allow for the blinding of participants. The only outcome measures employed in this study were self-reports, which introduces a high risk of bias due to the lack of participant blinding. Self-reported data are inherently vulnerable to various forms of bias, including social desirability, inaccurate self-perception, and response-shift bias, particularly when the intervention itself may influence participants’ interpretation of the measured behaviors [41,42]. While self-reports remain a practical method for assessing behavioral outcomes, their susceptibility to both random and systematic errors [43] underscores the need for cautious interpretation [44]. Future studies should consider incorporating objective indicators or triangulating multiple data sources to enhance the accuracy and validity of outcome assessments.

In addition, the lack of consistency within non-pharmacological interventions and the various assessment tools used to measure outcomes at inconsistent time points may have contributed to heterogeneity and limited the generalizability of the results. To address this, future research should aim to standardize intervention components and align follow-up assessment timings to allow for more robust comparisons across studies. Finally, this review included only English-language studies due to limited resources and the absence of translation support. As a result, relevant studies published in other languages may have been excluded, potentially introducing language bias and limiting the cultural and geographical diversity of the evidence base. This restriction may have also contributed to the heterogeneity observed across studies by narrowing the range of populations and contexts represented in the analysis. Future reviews should seek to include studies published in multiple languages to broaden the scope of evidence and enhance the global applicability of the findings.

## 5. Conclusions

This systematic review outlines the evidence on the effectiveness of web-based lifestyle improvement interventions for the preconception health of preconception women and those aiming to conceive. The findings from this review suggest that there may be a benefit from web-based interventions for blood pressure management, serum folate levels, and physical activity for preconception women. However, no significant benefits were shown for intake of vegetables and fruit, folic acid intake, smoking, alcohol consumption, waist circumference, weight, BMI, overweight status, HbA1c, total cholesterol, HDL, stress, depression, anxiety, or pregnancy outcomes. To address these gaps, future studies should aim to use sufficiently powered sample sizes to detect intervention effects, apply standardized and validated tools for assessing behavioral and clinical outcomes, and adopt theory-driven, structured intervention strategies. Therefore, this review suggests a need for high-quality studies to examine these preconception women from the perspective of improving food intake, folic acid intake, physical activity, smoking, and alcohol consumption habits. It is essential to clarify the need for preconception care as primary care at each stage of the intervention, with a view to future pregnancy outcomes and the early childhood health of the next generation.

## Figures and Tables

**Table 1:** PICO framework for the search strategy.

PICO Component	Description
Population	Reproductive-aged women or couples wishing to conceive, including those experiencing infertility or undergoing ART.
Intervention	Web-based or technology-assisted lifestyle interventions (e.g., eHealth, mHealth, mobile apps, telemedicine, digital education).
Comparison	Standard care or no intervention.
Outcomes	Food intake, folic acid intake, physical activity, smoking, alcohol consumption, stress management, and clinical and biochemical indicators.

**Table 2:** Characteristics of included studies.

Author/Year	Country/ Setting	Study Design	Participants	Sample Size	Intervention	Control	Evaluation Period	Outcomes
Hanafiah et al., 2022 [15]	Malaysia/district based	Individual RCT	Women aged 20–39 years; nulliparous; not pregnant;	549 women	Provided lifestyle challenges that the young couples could select, information on healthy lifestyles, and included a total of six contact points with community health promotors	Standard care provided by public health clinics	At 33 weeks from baseline	Clinical outcomes: waist circumstance; weight; BMI; blood pressure; HbA1c; total cholesterol; high-density lipoprotein (HDL); triglycerides; depression; anxiety; stress Lifestyle modification outcomes: Vegetable and Fruit intake; Proportion size of rice and bread; Frequency of fried Foods, fast food, carbonated drinks, pastries, and sweet local delicacies; Vigorous/Moderate job-related physical activity; Vigorous/Moderate leisure physical activity;
Jack et al., 2020 [16]	USA/ web based	Individual RCT	African American or Black or both; women aged 18–34 years; currently not pregnant	528 women	Visiting the Gabby character online and receiving an introductory engagement dialogue (log on at least once every 2 weeks)	Letter listing the risks identified and encouraging them to discuss these risks with a clinician	At 6 (24 w) and 12 (48 w) months after intervention	Clinical outcomes: anxiety; depression; stress; underweight; overweight Lifestyle modification outcomes: stages of change (SoC) [19] scores in the following: emotional and mental health (7 items); environmental issues (8 items); family planning (2 items); genetic health history (4 items); health care and programs (2 items); health conditions and medicine (17 items); immunizations and vaccines (8 items); infection diseases (12 items); men and health care (3 items); nutrition and activity (15 items); relationships (3 items); reproductive health (17 items); substance use (3 items)
Ng et al., 2021 [17]	UK/gynecological outpatient department	Individual RCT	Subfertility or recurrent miscarriage; women aged 18–45 years; actively trying to conceive	262 women	Personalized smartphone lifestyle coaching program and emails (maximum of three per week) with feedback on progress, recommendations, tips, facts, and recipes	Standard preconception advice	At 12 and 24 weeks after randomization	Clinical outcomes: pregnancy at 24 weeks after randomization Lifestyle modification outcomes: fruit intake; vegetable intake; taking folic acid supplements; smoking; alcohol
Oostingh et al., 2020 [18]	Netherlands/IVF centers	Individual RCT	Women aged 18–45 years; starting their IVF/ICSI treatment within the next 3 months	626 women	Coaching via smart phone based on sex, pregnancy status, and behaviors and monitoring changes in their identified risk behaviors and to assess pregnancy status	“Light” version of Smarter Pregnancy	At 24 weeks after completion of program	Clinical outcomes: serum folate levels of women; pregnancy rates at 52 weeks Lifestyle modification outcomes: vegetable intake; fruit intake; folic acid supplement use; dietary risk score; smoking; alcohol consumption; lifestyle risk score

**Table 3:** Outcomes reported in included studies.

	Outcomes	Hanafiah et al., 2022 [15]		Jack et al., 2020 [16]		Ng et al., 2021 [17]		Oostingh et al., 2020 [18]	
		Measurement	Results	Measurement	Results	Measurement	Results	Measurement	Results
Clinical outcomes	Anthropometric Indicators	Waist circumstance; weight; BMI; blood pressure (systolic); blood pressure (diastolic)	36 w: Waist circumstance: IG mean (SD); 82.6 (13.9) vs. CG mean (SD); 81.7 (13.9), *p* = 0.547. Weight: IG mean (SD); 66.7 (18.8) vs. CG mean (SD); 65.4 (17.5), *p* = 0.528. BMI: IG mean (SD) 27.3 (7.4) vs. CG mean (SD); 27.0 (7.0), *p* = 0.701. Blood pressure (systolic); IG mean (SD); 107.6 (14.0) vs. CG mean (SD); 104.7 (10.6), *p* = 0.031. Blood pressure (diastolic); IG mean (SD); 72.3 (10.5) vs. CG mean (SD); 70.9 (9.1), *p* = 0.230.	Underweight; overweight	Underweight: the data are unavailable due to missing data for the underweight group in the IG. Overweight: 24 w: IG mean; 3.85 vs. CG mean; 3.64, *p* = 0.30; 48 w: IG mean; 4.02 vs. CG mean; 4.05 *p* = 0.88.	Not reported		Not reported	
	Physiological and Biochemical Indicators	HbA1c; total cholesterol; high-density lipoprotein (HDL); triglycerides	36 w: HbA1c IG mean (SD); 5.3 (0.6) vs. CG mean (SD); 5.2 (0.5), *p* = 0.093. Total cholesterol; IG mean (SD); 4.86 (0.9) vs. CG mean (SD); 4.79 (0.9), *p* = 0.527. HDL: IG mean (SD); 1.45 (0.4) vs. CG mean (SD); 1.43 (0.4), *p* = 0.632.	Not reported		Not reported		Serum folate levels of women	12 w: IG median 48.6 (IQR 28.8–64.1) nmol/L vs. CG median 30.1 (IQR 17.9–51.9) nmol/L
	Mental Health Status	Stress, moderate or severe (DASS-21) [19]	36 w: moderate; IG; 15.9% vs. CG; 16.3%, severe; IG; 2.1% vs. CG; 6.3%, *p* = 0.19.	Stress (PSS)	24 w: IG mean; 4.04 vs. CG mean; 3.60, *p* = 0.21. 48 w: IG mean; 4.09 vs. CG mean; 3.89, *p* = 0.52.	Not reported		Not reported	
		Depression, moderate or severe (DASS-21) [19]	36 w: moderate; IG; 34.5% vs. CG; 33.1%, severe; IG; 2% vs. CG; 6%, *p* = 0.64	Potential Depression (PHQ-2)	24 w: IG mean; 3 vs. CG mean; 2.77, *p* = 0.66. 48 w: IG mean; 3.27 vs. CG mean; 3.58, *p* = 0.41.	Not reported		Not reported	
		Anxiety moderate or severe (DASS-21) [19]	36 w: moderate; IG; 20.0% vs. CG; 19.4%, severe; IG; 1.4% vs. CG; 3.1%, *p* = 0.60.	Not reported		Not reported		Not reported	
	Pregnancy-related outcomes	Not reported		Not reported		Pregnancy	24 w: difference between IG and CG; 2.83 (95% CI 0.35 to 57.76)	Pregnancy	52w: difference between IG and CG; 0.807 (95% CI 0.574 to 1.134)
Lifestyle modification outcomes	Vegetable intake	Vegetable intake/week	36 w: IG mean ± SD; 9.4 ± 7.8 vs. CG mean ± SD; 8.7 ± 6.1, *p* = 0.458.	SoC score of bad diet or food choices (<5 daily servings of fruits and vegetables and/or regular intake of junk food)	24 w: IG mean; 3.53 vs. CG mean; 3.27, *p* = 0.27. 48 w: IG mean; 3.55 vs. CG mean; 3.39, *p* = 0.57.	Vegetable intake risk score (“0” adequate to “3” inadequate)	12 w: difference between IG and CG; −0.21 (95% CI −0.48 to 0.03) 24 w: difference between IG and CG; 0.00 (95% CI −0.30 to 0.27)	number of vegetables > 200 g/day	24 w: IG; 40% vs. CG; 28% 36 w: IG; 33% vs. CG; 21%
	Fruit intake	Fruit intake/week	36 w: IG mean ± SD; 5.8 ± 4.8 vs. CG mean ± SD; 5.1 ± 4.2, *p* = 0.199.	SoC score of bad diet or food choices (<5 daily servings of fruits and vegetables and/or regular intake of junk food)	24 w: IG mean; 3.53 vs. CG mean; 3.27, *p* = 0.27. 48 w: IG mean; 3.55 vs. CG mean; 3.39, *p* = 0.57.	Fruit intake risk score (“0” adequate to “3” inadequate)	12 w: difference between IG and CG; −0.14 (95% CI −0.60 to 0.07) 24 w: difference between IG and CG; −0.21 (95% CI −0.50 to 0.66)	Fruits, >2 pieces per day	24 w: IG; 67% vs. CG; 44% 36 w: IG; 68% vs. CG; 57%
	Folic acid intake	Not reported		SoC score of not using multivitamin with folic acid or folic acid supplement	24 w: IG mean; 3.29 vs. CG mean; 3.13, *p* = 0.47. 48 w: IG mean; 3.45 vs. CG mean; 3.32, *p* = 0.61.	Taking folic acid supplements (“0” adequate to “3” inadequate)	12 w: difference between IG and CG; −0.04 (95% CI −0.29 to 0.21) 24 w: difference between IG and CG; −0.16 (95% CI −0.42 to 0.09)	Taking adequate folic acid supplement	24 w: IG; 97% vs. CG; 99% 36 w: IG; 96% vs. CG; 96%
	Smoking	Not reported		SoC score of any current tobacco use	24 w: IG mean; 2.46 vs. CG mean; 2.38, *p* = 0.90. 48 w: IG mean; 3.54 vs. CG mean; 3.4, *p* = 0.89.	Smoking risk score (“0” no smoking to “6” 15 or more cigarettes/day)	12 w: difference between IG and CG; 0.02 (95% CI −0.01 to 0.10) 24 w: difference between IG and CG; 0.08 (95% CI −0.02 to 0.28)	No smoking	24 w: IG; 93% vs. CG; 92% 36 w: IG; 80% vs. CG; 91%
	Alcohol consumption	Not reported		SoC score of excessive alcohol (≥4 drinks in a day over the past year)	24 w: IG mean; 3.45 vs. CG mean; 3.67, *p* = 0.50. 48 w: IG mean; 4.03 vs. CG mean; 3.54, *p* = 0.13.	Alcohol risk score (“0” no alcohol intake to “3” 3 or more alcohol beverages/day)	12 w: difference between IG and CG; 0.0 (95% CI −0.14 to 0.09) 24 w: difference between IG and CG; −0.02 (95% CI −0.15 to 0.10)	No alcohol consumption	24 w: IG; 78% vs. CG; 73% 36 w: IG; 69% vs. CG; 75%
				SoC score of excessive alcohol (drinking more than twice a week)	24 w: IG mean; 3.69 vs. CG mean; 3.74, *p* = 0.88. 48 w: IG mean; 4.09 vs. CG mean; 3.66, *p* = 0.19.				
	Physical Activity	Vigorous job-related physical activity, mins/week	36 w: IG mean ± SD; 259.9 ± 389.7 vs. CG mean ± SD; 153.8 ± 280.2, *p* = 0.032	SoC score of not enough exercise	24 w: IG mean; 3.25 vs. CG mean; 3.35, *p* = 0.60. 48 w: IG mean; 3.50 vs. CG mean; 3.51, *p* = 0.97.	Not reported		Not reported	
		Moderate job-related physical activity, mins/week	36 w: IG mean ± SD; 749.0 ± 822.0 vs. CG mean ± SD; 550.0 ± 725.4, *p* = 0.058.						
		Vigorous leisure physical activity, mins/week	36 w: IG mean ± SD; 120.5 ± 143.6 vs. CG mean ± SD; 138.5 ± 164.7, *p* = 0.417.						
		Moderate leisure physical activity, mins/week	36 w: IG mean ± SD; 271.8 ± 463.2 vs. CG mean ± SD; 328.6 ± 607.0, *p* = 0.434.						

Note: SoC—stage of change; IG—intervention group; CG—control group.

## Data Availability

Data are contained within the article.

## Supplementary Materials

The following supporting information can be downloaded at: https://www.mdpi.com/article/10.3390/healthcare13091037/s1, File S1: Search Strategy and File S2: List of Excluded Full Text Articles.
