# Supplementary material for: Effectiveness of Web-Based Interventions on Clinical Outcomes and Lifestyle Modifications in Women Planning to Conceive: A Systematic Review
Source: Healthcare (Basel). 2025 May 1;13(9):1037. doi: 10.3390/healthcare13091037 (PMC12072171; doi:10.3390/healthcare13091037)
Supplement: Supplementary file 1 [file healthcare-13-01037-s001.zip › healthcare-3534044-supplementary.pdf]

Supplementary file

Supplement to: Suzuki et al. Effectiveness of Web-based Interventions on Clinical Outcomes and Lifestyle Modifications in Women Planning to Conceive: A Systematic Review

S1. Search strategies

CENTRAL

(("health education" OR "health promotion" OR "self care" OR "literacy" OR "health literacy" OR "behavior therapy" OR "psychosocial intervention" OR "mentoring" OR "mentoring" OR "smartphone" OR "cell phone" OR "computers" OR "telemedicine" OR "internet of things" OR "nutritional support" OR "nutrition therapy" OR "nutritional status" OR "eating" OR "diet" OR "malnutrition" OR "nutrition disorders" OR "overweight" OR "obesity" OR "energy intake" OR "Text Messaging" OR "Social Media" OR "non drug interventions" OR "non pharmacological interventions" OR "non drug intervention" OR "non pharmacological intervention" OR "nondrug interventions" OR "nonpharmacological interventions" OR "nondrug intervention" OR "nonpharmacological intervention" OR "health education" OR "health promotion" OR "program" OR "programs" OR "programme" OR "programmes" OR "campaign" OR "campaigns" OR "health knowledge" OR "lifestyle guidance" OR "life style guidance" OR "health consultation" OR "consultation services" OR "consultation service" OR "selfcare" OR "self care" OR "self management" OR "literacy" OR "health literacy" OR "lifestyle intervention" OR "lifestyle interventions" OR "lifestyle improvement" OR "lifestyle improvements" OR "lifestyle modification" OR "lifestyle modification" OR "life style intervention" OR "life style interventions" OR "life style improvement" OR "life style improvements" OR "life style modification" OR "life style modification" OR "behavioral therapy" OR "behavioural therapy" OR "psychosocial intervention" OR "psychosocial interventions" OR "health checkup" OR "health checkups" OR "medical checkup" OR "medical checkup" OR "lifestyle advice" OR "coaching" OR "coach" OR "coaches" OR "coached" OR "smart phone" OR "smart phones" OR "mobile phone" OR "mobile phones" OR "computer" OR "computers" OR "tablet" OR "tablets" OR "tele health" OR "mHealth" OR "eHealth" OR "web" OR "website" OR "social media" OR "twitter" OR "facebook" OR "youtube" OR "network" OR "networks" OR "chat" OR "internet of things" OR "nutrition therapy" OR "nutritional intervention" OR "nutritional interventions" OR "food intake" OR "eating" OR "diet" OR "diets" OR "dieting" OR "nutrition" OR "malnutrition" OR "nutrition disorders" OR "nutrition disorder" OR "malnourishment" OR "undernutrition" OR "overweight" OR "obesity" OR "obese" OR "nutrition assessment" OR "nutritional assessment" OR "nutritional status" OR "nutrition status" OR "energy intake" OR "body mass index" OR "BMI" OR "nutritional support" OR "nutrition support" OR "SMS" OR "text message" OR "text messages" OR "text messaging" OR "texting" OR "Internet" OR "information technology" OR ("intake" AND ("fruits" OR "fruit" OR "vegetable" OR "vegetables" OR "folic acid")) OR smoking OR "alcohol use" OR "alcohol drinking") AND ("Life Style" OR "Life Style" OR "Life Styles" OR "Lifestyle" OR "Lifestyles" OR "Healthy Lifestyle" OR "Health Behavior" OR "Health Behavior" OR "Health Behaviors" OR "Health Behaviour" OR "Health Behaviours" OR "guidance" OR "Preconception Care" OR "preconception care") AND

("infertility" OR "infertile" OR "subfertility" OR "subfertility" OR "preconception period" OR "periconception period" OR "Reproductive Techniques" OR "Assisted Reproduction" OR "Donor Conception" OR "Embryo Transfer" OR "Fertility Preservation" OR "Fertilization in Vitro" OR "Mitochondrial Replacement Therapy" OR "Intracytoplasmic Sperm Injections" OR "Intracytoplasmic Sperm Injection" OR "Gamete Intrafallopian Transfer" OR "In Vitro Oocyte Maturation" OR "Artificial Insemination" OR "Oocyte Donation" OR "Oocyte Retrieval" OR "Ovulation Induction" OR "Superovulation" OR "Posthumous Conception" OR "Sperm Retrieval" OR "Zygote Intrafallopian Transfer" OR (("conceive" OR "conception") AND (wish OR wishes OR wishing OR wished))) AND ("Female" OR "Women" OR "female" OR "females" OR "women" OR "woman" OR "girl" OR "girls" OR "couple" OR "couples")):ti,ab,kw

PubMed

(("health education"[mesh] OR "health promotion"[mesh] OR "self care"[mesh] OR "literacy"[mesh] OR "health literacy"[mesh] OR "behavior therapy"[mesh] OR "psychosocial intervention"[mesh] OR "mentoring"[mesh] OR "mentoring"[tw] OR "smartphone"[mesh] OR "cell phone"[mesh] OR "computers"[mesh] OR "telemedicine"[mesh] OR "internet of things"[mesh] OR "nutritional support"[mesh] OR "nutrition therapy"[mesh] OR "nutritional status"[mesh] OR "eating"[mesh] OR "diet"[mesh] OR "malnutrition"[mesh] OR "nutrition disorders"[mesh] OR "overweight"[mesh] OR "obesity"[mesh] OR "energy intake"[mesh] OR "Text Messaging"[Mesh] OR "Social Media"[Mesh] OR "Internet"[mesh] OR "non-drug interventions"[tw] OR "non-pharmacological interventions"[tw] OR "non-drug intervention"[tw] OR "non-pharmacological intervention"[tw] OR "nonpharmacological intervention"[tw] OR "nonpharmacological interventions"[tw] OR "non-pharmacologic interventions"[tw] OR "non-pharmacologic intervention"[tw] OR "nonpharmacologic interventions"[tw] OR "nonpharmacologic intervention"[tw] OR "health education"[tw] OR "health promotion"[tw] OR "program"[tw] OR "programs"[tw] OR "programme"[tw] OR "programmes"[tw] OR "campaign"[tw] OR "campaigns"[tw] OR "health knowledge"[tw] OR "lifestyle guidance"[tw] OR "life style guidance"[tw] OR "health consultation"[tw] OR "consultation services"[tw] OR "consultation service"[tw] OR "selfcare"[tw] OR "self care"[tw] OR "self management"[tw] OR "literacy"[tw] OR "health literacy"[tw] OR "lifestyle intervention"[tw] OR "lifestyle interventions"[tw] OR "lifestyle improvement"[tw] OR "lifestyle improvements"[tw] OR "lifestyle modification"[tw] OR "lifestyle modification"[tw] OR "life style intervention"[tw] OR "life style interventions"[tw] OR "life style improvement"[tw] OR "life style improvements"[tw] OR "life style modification"[tw] OR "life style modification"[tw] OR "behavioral therapy"[tw] OR "behavioural therapy"[tw] OR "psychosocial intervention"[tw] OR "psychosocial interventions"[tw] OR "health checkup"[tw] OR "health checkups"[tw] OR "medical checkup"[tw] OR "medical checkup"[tw] OR "lifestyle advice"[tw] OR "coaching"[tw] OR "coach"[tw] OR "coaches"[tw] OR "coached"[tw] OR "smart phone"[tw] OR "smart phones"[tw] OR "mobile phone"[tw] OR "mobile phones"[tw] OR "computer"[tw] OR "computers"[tw] OR "tablet"[tw] OR "tablets"[tw] OR "tele health"[tw] OR "mHealth"[tw] OR "eHealth"[tw] OR "web"[tw]

OR "website"[tw] OR "social media"[tw] OR "twitter"[tw] OR "facebook"[tw] OR "youtube"[tw] OR "network"[tw] OR "networks"[tw] OR "chat"[tw] OR "internet of things"[tw] OR "nutrition therapy"[tw] OR "nutritional intervention"[tw] OR "nutritional interventions"[tw] OR "food intake"[tw] OR "eating"[tw] OR "diet"[tw] OR "diets"[tw] OR "dieting"[tw] OR "nutrition"[tw] OR "malnutrition"[tw] OR "nutrition disorders"[tw] OR "nutrition disorder"[tw] OR "malnourishment"[tw] OR "undernutrition"[tw] OR "overweight"[tw] OR "obesity"[tw] OR "obese"[tw] OR "nutrition assessment"[tw] OR "nutritional assessment"[tw] OR "nutritional status"[tw] OR "nutrition status"[tw] OR "energy intake"[tw] OR "body mass index"[tw] OR "BMI"[tw] OR "nutritional support"[tw] OR "nutrition support"[tw] OR "SMS"[tw] OR "text message"[tw] OR "text messages"[tw] OR "text messaging"[tw] OR "texting"[tw] OR "Internet"[tw] OR "information technology"[tw] OR ("intake"[tw] AND ("fruits"[tw] OR "fruit"[tw] OR "vegetable"[tw] OR "vegetables"[tw] OR "folic acid"[tw])) OR smoking[tw] OR "alcohol use"[tw] OR "alcohol drinking"[tw]) AND ("Life Style"[Mesh] OR "Life Style"[tw] OR "Life Styles"[tw] OR "Lifestyle"[tw] OR "Lifestyles"[tw] OR "Healthy Lifestyle"[Mesh] OR "Health Behavior"[mesh] OR "Health Behavior"[tw] OR "Health Behaviors"[tw] OR "Health Behaviour"[tw] OR "Health Behaviours"[tw] OR "guidance"[tw] OR "Preconception Care"[mesh] OR "preconception care"[tw]) AND ("Infertility, Female"[mesh] OR "infertility"[tw] OR "infertile"[tw] OR "subfertility"[tw] OR "subfertility"[tw] OR "preconception period"[tw] OR "periconception period"[tw] OR "Reproductive Techniques, Assisted"[Mesh] OR "Assisted Reproduction"[tw] OR "Donor Conception"[tw] OR "Embryo Transfer"[tw] OR "Fertility Preservation"[tw] OR "Fertilization in Vitro"[tw] OR "Mitochondrial Replacement Therapy"[tw] OR "Intracytoplasmic Sperm Injections"[tw] OR "Intracytoplasmic Sperm Injection"[tw] OR "Gamete Intrafallopian Transfer"[tw] OR "In Vitro Oocyte Maturation"[tw] OR "Artificial Insemination"[tw] OR "Oocyte Donation"[tw] OR "Oocyte Retrieval"[tw] OR "Ovulation Induction"[tw] OR "Superovulation"[tw] OR "Posthumous Conception"[tw] OR "Sperm Retrieval"[tw] OR "Zygote Intrafallopian Transfer"[tw] OR "wish conceive"[title/abstract:~4] OR "wish conception"[title/abstract:~4] OR "wishes conceive"[title/abstract:~4] OR "wishes conception"[title/abstract:~4] OR "wishing conceive"[title/abstract:~4] OR "wishing conception"[title/abstract:~4]) AND ("Female"[mesh] OR "Women"[mesh] OR "female"[tw] OR "females"[tw] OR "women"[tw] OR "woman"[tw] OR "girl"[tw] OR "girls"[tw] OR "couple"[tw] OR "couples"[tw]) AND ("clinical trial"[pt] OR "clinical trial"[tw] OR "clinical trials as topic"[mesh] OR "clinical trials"[tw] OR "control groups"[mesh] OR "control group"[tw] OR "control groups"[tw] OR "controlled clinical trial"[pt] OR "controlled clinical trials as topic"[mesh] OR "cross-over studies"[mesh] OR "cross over study"[tw] OR "cross over studies"[tw] OR "double-blind method"[mesh] OR "double blind"[tw] OR "evaluation studies as topic"[mesh] OR "follow-up studies"[mesh] OR "follow up study"[tw] OR "follow up studies"[tw] OR "placebos"[mesh] OR placebo*[tw] OR placebos*[tw] OR "pragmatic clinical trial"[pt] OR "prospective studies"[mesh] OR "prospective study"[tw] OR "prospective studies"[tw] OR "RaCT"[tw] OR "RaCTs"[tw] OR "random allocation"[mesh] OR "randomised "[tw] OR "randomized controlled trial"[pt] OR "randomized controlled trials as topic"[mesh] OR "randomized"[tw] OR random*[tw] OR "RCT"[tw] OR "RCTs"[tw] OR "Research Design"[MeSH:noexp] OR "Research design"[tw] OR "Research designs"[tw] OR "single blind"[tw] OR "single-

blind method"[mesh] OR ((single*[tw] OR double*[tw] OR triple*[tw]) AND (blind*[tw] OR mask*[tw])) OR volunteer*[tw] OR "trial"[ti] OR "trials"[ti]) NOT ("Animals"[mesh] NOT "Humans"[mesh]))

Web of Science

(TS=("health education" OR "health promotion" OR "self care" OR "literacy" OR "health literacy" OR "behavior therapy" OR "psychosocial intervention" OR "mentoring" OR "mentoring" OR "smartphone" OR "cell phone" OR "computers" OR "telemedicine" OR "internet of things" OR "nutritional support" OR "nutrition therapy" OR "nutritional status" OR "eating" OR "diet" OR "malnutrition" OR "nutrition disorders" OR "overweight" OR "obesity" OR "energy intake" OR "Text Messaging" OR "Social Media" OR "non drug interventions" OR "non pharmacological interventions" OR "non drug intervention" OR "non pharmacological intervention" OR "nondrug interventions" OR "nonpharmacological interventions" OR "nondrug intervention" OR "nonpharmacological intervention" OR "health education" OR "health promotion" OR "program" OR "programs" OR "programme" OR "programmes" OR "campaign" OR "campaigns" OR "health knowledge" OR "lifestyle guidance" OR "life style guidance" OR "health consultation" OR "consultation services" OR "consultation service" OR "selfcare" OR "self care" OR "self management" OR "literacy" OR "health literacy" OR "lifestyle intervention" OR "lifestyle interventions" OR "lifestyle improvement" OR "lifestyle improvements" OR "lifestyle modification" OR "lifestyle modification" OR "life style intervention" OR "life style interventions" OR "life style improvement" OR "life style improvements" OR "life style modification" OR "life style modification" OR "behavioral therapy" OR "behavioural therapy" OR "psychosocial intervention" OR "psychosocial interventions" OR "health checkup" OR "health checkups" OR "medical checkup" OR "medical checkup" OR "lifestyle advice" OR "coaching" OR "coach" OR "coaches" OR "coached" OR "smart phone" OR "smart phones" OR "mobile phone" OR "mobile phones" OR "computer" OR "computers" OR "tablet" OR "tablets" OR "tele health" OR "mHealth" OR "eHealth" OR "web" OR "website" OR "social media" OR "twitter" OR "facebook" OR "youtube" OR "network" OR "networks" OR "chat" OR "internet of things" OR "nutrition therapy" OR "nutritional intervention" OR "nutritional interventions" OR "food intake" OR "eating" OR "diet" OR "diets" OR "dieting" OR "nutrition" OR "malnutrition" OR "nutrition disorders" OR "nutrition disorder" OR "malnourishment" OR "undernutrition" OR "overweight" OR "obesity" OR "obese" OR "nutrition assessment" OR "nutritional assessment" OR "nutritional status" OR "nutrition status" OR "energy intake" OR "body mass index" OR "BMI" OR "nutritional support" OR "nutrition support" OR "SMS" OR "text message" OR "text messages" OR "text messaging" OR "texting" OR "Internet" OR "information technology" OR ("intake" AND ("fruits" OR "fruit" OR "vegetable" OR "vegetables" OR "folic acid")) OR smoking OR "alcohol use" OR "alcohol drinking") AND TS=("Life Style" OR "Life Style" OR "Life Styles" OR "Lifestyle" OR "Lifestyles" OR "Healthy Lifestyle" OR "Health Behavior" OR "Health Behavior" OR "Health Behaviors" OR "Health Behaviour" OR "Health Behaviours" OR "guidance" OR "Preconception Care" OR "preconception care") AND TS=("infertility" OR "infertile" OR "subfertility" OR "subfertility" OR "preconception

period" OR "periconception period" OR "Reproductive Techniques" OR "Assisted Reproduction" OR "Donor Conception" OR "Embryo Transfer" OR "Fertility Preservation" OR "Fertilization in Vitro" OR "Mitochondrial Replacement Therapy" OR "Intracytoplasmic Sperm Injections" OR "Intracytoplasmic Sperm Injection" OR "Gamete Intrafallopian Transfer" OR "In Vitro Oocyte Maturation" OR "Artificial Insemination" OR "Oocyte Donation" OR "Oocyte Retrieval" OR "Ovulation Induction" OR "Superovulation" OR "Posthumous Conception" OR "Sperm Retrieval" OR "Zygote Intrafallopian Transfer" OR (("conceive" OR "conception") AND (wish OR wishes OR wishing OR wished))) AND TS=("Female" OR "Women" OR "female" OR "females" OR "women" OR "woman" OR "girl" OR "girls" OR "couple" OR "couples") AND (TS=("clinical trial" OR "clinical trial" OR "clinical trials as topic" OR "clinical trials" OR "control groups" OR "control group" OR "control groups" OR "controlled clinical trial" OR "controlled clinical trials as topic" OR "cross-over studies" OR "cross over study" OR "cross over studies" OR "double-blind method" OR "double blind" OR "evaluation studies as topic" OR "follow-up studies" OR "follow up study" OR "follow up studies" OR "placebos" OR placebo* OR placebos* OR "pragmatic clinical trial" OR "prospective studies" OR "prospective study" OR "prospective studies" OR "RaCT" OR "RaCTs" OR "random allocation" OR "randomised " OR "randomized controlled trial" OR "randomized controlled trials as topic" OR "randomized" OR random* OR "RCT" OR "RCTs" OR "Research Design" OR "Research design" OR "Research designs" OR "single blind" OR "single-blind method" OR ((single* OR double* OR triple*) AND (blind* OR mask*)) OR volunteer*) OR TI=("trial" OR "trials") OR AK=("trial" OR "trials")) NOT ti=("veterinary" OR "rabbit" OR "rabbits" OR "animal" OR "animals" OR "mouse" OR "mice" OR "rodent" OR "rodents" OR "rat" OR "rats" OR "pig" OR "pigs" OR "porcine" OR "horse" OR "horses" OR "equine" OR "cow" OR "cows" OR "bovine" OR "goat" OR "goats" OR "sheep" OR "ovine" OR "canine" OR "dog" OR "dogs" OR "feline" OR "cat" OR "cats"))

Embase (OVID version)

((exp "health education"/ OR exp "health promotion"/ OR exp "self care"/ OR exp "literacy"/ OR exp "health literacy"/ OR exp "behavior therapy"/ OR exp "psychosocial intervention"/ OR exp "mentoring"/ OR exp "smartphone"/ OR exp "cell phone"/ OR exp "computer"/ OR exp "telemedicine"/ OR exp "internet of things"/ OR exp "nutritional support"/ OR exp "nutrition therapy"/ OR exp "nutritional status"/ OR exp "eating"/ OR exp "diet"/ OR exp "malnutrition"/ OR exp "nutrition disorder"/ OR exp "overweight"/ OR exp "obesity"/ OR exp "energy intake"/ OR exp "Text Messaging"/ OR exp "Social Media"/ OR exp "Internet"/ OR "mentoring".mp OR "non-drug interventions".mp OR "non-pharmacological interventions".mp OR "non-drug intervention".mp OR "non-pharmacological intervention".mp OR "health education".mp OR "health promotion".mp OR "program".mp OR "programs".mp OR "programme".mp OR "programmes".mp OR "campaign".mp OR "campaigns".mp OR "health knowledge".mp OR "lifestyle guidance".mp OR "life style guidance".mp OR "health consultation".mp OR "consultation services".mp OR "consultation service".mp OR "selfcare".mp OR "self care".mp OR "self management".mp OR "literacy".mp OR "health literacy".mp OR

"lifestyle intervention".mp OR "lifestyle interventions".mp OR "lifestyle improvement".mp OR "lifestyle improvements".mp OR "lifestyle modification".mp OR "lifestyle modification".mp OR "life style intervention".mp OR "life style interventions".mp OR "life style improvement".mp OR "life style improvements".mp OR "life style modification".mp OR "life style modification".mp OR "behavioral therapy".mp OR "behavioural therapy".mp OR "psychosocial intervention".mp OR "psychosocial interventions".mp OR "health checkup".mp OR "health checkups".mp OR "medical checkup".mp OR "medical checkup".mp OR "lifestyle advice".mp OR "coaching".mp OR "coach".mp OR "coaches".mp OR "coached".mp OR "smart phone".mp OR "smart phones".mp OR "mobile phone".mp OR "mobile phones".mp OR "computer".mp OR "computers".mp OR "tablet".mp OR "tablets".mp OR "tele health".mp OR "mHealth".mp OR "eHealth".mp OR "web".mp OR "website".mp OR "social media".mp OR "twitter".mp OR "facebook".mp OR "youtube".mp OR "network".mp OR "networks".mp OR "chat".mp OR "internet of things".mp OR "nutrition therapy".mp OR "nutritional intervention".mp OR "nutritional interventions".mp OR "food intake".mp OR "eating".mp OR "diet".mp OR "diets".mp OR "dieting".mp OR "nutrition".mp OR "malnutrition".mp OR "nutrition disorders".mp OR "nutrition disorder".mp OR "malnourishment".mp OR "undernutrition".mp OR "overweight".mp OR "obesity".mp OR "obese".mp OR "nutrition assessment".mp OR "nutritional assessment".mp OR "nutritional status".mp OR "nutrition status".mp OR "energy intake".mp OR "body mass index".mp OR "BMI".mp OR "nutritional support".mp OR "nutrition support".mp OR "SMS".mp OR "text message".mp OR "text messages".mp OR "text messaging".mp OR "texting".mp OR "information technology".mp OR ("intake".mp AND ("fruits".mp OR "fruit".mp OR "vegetable".mp OR "vegetables".mp OR "folic acid".mp)) OR smoking.mp OR "alcohol use".mp OR "alcohol drinking".mp) AND (exp "Lifestyle"/ OR "Life Style".mp OR "Life Styles".mp OR "Lifestyle".mp OR "Lifestyles".mp OR "Healthy Lifestyle"/ OR exp "Health Behavior"/ OR "Health Behavior".mp OR "Health Behaviors".mp OR "Health Behaviour".mp OR "Health Behaviours".mp OR "guidance".mp OR "Preconception Care"/ OR "preconception care".mp) AND (exp "Female Infertility"/ OR "infertility".mp OR "infertile".mp OR "subfertility".mp OR "subfertility".mp OR "preconception period".mp OR "periconception period".mp OR exp "Infertility therapy"/ OR "Assisted Reproduction".mp OR "Donor Conception".mp OR "Embryo Transfer".mp OR "Fertility Preservation".mp OR "Fertilization in Vitro".mp OR "Mitochondrial Replacement Therapy".mp OR "Intracytoplasmic Sperm Injections".mp OR "Intracytoplasmic Sperm Injection".mp OR "Gamete Intrafallopian Transfer".mp OR "In Vitro Oocyte Maturation".mp OR "Artificial Insemination".mp OR "Oocyte Donation".mp OR "Oocyte Retrieval".mp OR "Ovulation Induction".mp OR "Superovulation".mp OR "Posthumous Conception".mp OR "Sperm Retrieval".mp OR "Zygote Intrafallopian Transfer".mp OR (("wish" ADJ4 "conceive") OR ("wish" ADJ4 "conception") OR ("wishes" ADJ4 "conceive") OR ("wishes" ADJ4 "conception") OR ("wishing" ADJ4 "conceive") OR ("wishing" ADJ4 "conception")).mp) AND (exp "Female"/ OR exp "Women"/ OR "female".mp OR "females".mp OR "women".mp OR "woman".mp OR "girl".mp OR "girls".mp OR "couple".mp OR "couples".mp) AND (exp randomized controlled trial/ OR random*.ti,ab OR ((pragmatic OR practical) adj clinical trial*).ti,ab OR ((non-inferiority OR noninferiority OR superiority OR equivalence) adj3 trial*).ti,ab) NOT ((exp animal/ OR exp "animal experiment"/ OR exp "animal model"/

OR exp "nonhuman"/) NOT exp "human"/) NOT (conference review OR editorial OR letter OR note).pt)

Emcare (OVID version)

((exp "health education"/ OR exp "health promotion"/ OR exp "self care"/ OR exp "literacy"/ OR exp "health literacy"/ OR exp "behavior therapy"/ OR exp "psychosocial intervention"/ OR exp "mentoring"/ OR exp "smartphone"/ OR exp "cell phone"/ OR exp "computer"/ OR exp "telemedicine"/ OR exp "internet of things"/ OR exp "nutritional support"/ OR exp "nutrition therapy"/ OR exp "nutritional status"/ OR exp "eating"/ OR exp "diet"/ OR exp "malnutrition"/ OR exp "nutrition disorder"/ OR exp "overweight"/ OR exp "obesity"/ OR exp "energy intake"/ OR exp "Text Messaging"/ OR exp "Social Media"/ OR exp "Internet"/ OR "mentoring".mp OR "non-drug interventions".mp OR "non-pharmacological interventions".mp OR "non-drug intervention".mp OR "non-pharmacological intervention".mp OR "health education".mp OR "health promotion".mp OR "program".mp OR "programs".mp OR "programme".mp OR "programmes".mp OR "campaign".mp OR "campaigns".mp OR "health knowledge".mp OR "lifestyle guidance".mp OR "life style guidance".mp OR "health consultation".mp OR "consultation services".mp OR "consultation service".mp OR "selfcare".mp OR "self care".mp OR "self management".mp OR "literacy".mp OR "health literacy".mp OR "lifestyle intervention".mp OR "lifestyle interventions".mp OR "lifestyle improvement".mp OR "lifestyle improvements".mp OR "lifestyle modification".mp OR "lifestyle modification".mp OR "life style intervention".mp OR "life style interventions".mp OR "life style improvement".mp OR "life style improvements".mp OR "life style modification".mp OR "life style modification".mp OR "behavioral therapy".mp OR "behavioural therapy".mp OR "psychosocial intervention".mp OR "psychosocial interventions".mp OR "health checkup".mp OR "health checkups".mp OR "medical checkup".mp OR "medical checkup".mp OR "lifestyle advice".mp OR "coaching".mp OR "coach".mp OR "coaches".mp OR "coached".mp OR "smart phone".mp OR "smart phones".mp OR "mobile phone".mp OR "mobile phones".mp OR "computer".mp OR "computers".mp OR "tablet".mp OR "tablets".mp OR "tele health".mp OR "mHealth".mp OR "eHealth".mp OR "web".mp OR "website".mp OR "social media".mp OR "twitter".mp OR "facebook".mp OR "youtube".mp OR "network".mp OR "networks".mp OR "chat".mp OR "internet of things".mp OR "nutrition therapy".mp OR "nutritional intervention".mp OR "nutritional interventions".mp OR "food intake".mp OR "eating".mp OR "diet".mp OR "diets".mp OR "dieting".mp OR "nutrition".mp OR "malnutrition".mp OR "nutrition disorders".mp OR "nutrition disorder".mp OR "malnourishment".mp OR "undernutrition".mp OR "overweight".mp OR "obesity".mp OR "obese".mp OR "nutrition assessment".mp OR "nutritional assessment".mp OR "nutritional status".mp OR "nutrition status".mp OR "energy intake".mp OR "body mass index".mp OR "BMI".mp OR "nutritional support".mp OR "nutrition support".mp OR "SMS".mp OR "text message".mp OR "text messages".mp OR "text messaging".mp OR "texting".mp OR "information technology".mp OR ("intake".mp AND ("fruits".mp OR "fruit".mp OR "vegetable".mp OR "vegetables".mp OR "folic acid".mp)) OR smoking.mp OR "alcohol use".mp OR "alcohol drinking".mp OR ("intake".mp AND

("fruits".mp OR "fruit".mp OR "vegetable".mp OR "vegetables".mp OR "folic acid".mp)) OR smoking.mp OR "alcohol use".mp OR "alcohol drinking".mp) AND (exp "Lifestyle"/ OR "Life Style".mp OR "Life Styles".mp OR "Lifestyle".mp OR "Lifestyles".mp OR "Healthy Lifestyle"/ OR exp "Health Behavior"/ OR "Health Behavior".mp OR "Health Behaviors".mp OR "Health Behaviour".mp OR "Health Behaviours".mp OR "guidance".mp OR "Preconception Care"/ OR "preconception care".mp) AND (exp "Female Infertility"/ OR "infertility".mp OR "infertile".mp OR "subfertility".mp OR "subfertility".mp OR "preconception period".mp OR "periconception period".mp OR exp "Infertility therapy"/ OR "Assisted Reproduction".mp OR "Donor Conception".mp OR "Embryo Transfer".mp OR "Fertility Preservation".mp OR "Fertilization in Vitro".mp OR "Mitochondrial Replacement Therapy".mp OR "Intracytoplasmic Sperm Injections".mp OR "Intracytoplasmic Sperm Injection".mp OR "Gamete Intrafallopian Transfer".mp OR "In Vitro Oocyte Maturation".mp OR "Artificial Insemination".mp OR "Oocyte Donation".mp OR "Oocyte Retrieval".mp OR "Ovulation Induction".mp OR "Superovulation".mp OR "Posthumous Conception".mp OR "Sperm Retrieval".mp OR "Zygote Intrafallopian Transfer".mp OR (("wish" ADJ4 "conceive") OR ("wish" ADJ4 "conception") OR ("wishes" ADJ4 "conceive") OR ("wishes" ADJ4 "conception") OR ("wishing" ADJ4 "conceive") OR ("wishing" ADJ4 "conception")).mp) AND (exp "Female"/ OR exp "Women"/ OR "female".mp OR "females".mp OR "women".mp OR "woman".mp OR "girl".mp OR "girls".mp OR "couple".mp OR "couples".mp) AND (exp randomized controlled trial/ OR random*.ti,ab OR ((pragmatic OR practical) adj clinical trial*).ti,ab OR ((non-inferiority OR noninferiority OR superiority OR equivalence) adj3 trial*).ti,ab) NOT ((exp animal/ OR exp "animal experiment"/ OR exp "animal model"/ OR exp "nonhuman"/) NOT exp "human"/) NOT (editorial OR letter OR note).pt)

WHO International Clinical Trials Registry Platform

("Life Style" OR "Lifestyle" OR "Health Behavior" OR "Health Behaviour" OR "Preconception Care") AND ("Female" OR "Women" OR "females" OR "woman" OR "couple" OR "couples") AND ("infertility" OR "infertile" OR "subfertility" OR "subfertility" OR "preconception period" OR "periconception period" OR "Reproductive Techniques" OR "Assisted Reproduction" OR "Donor Conception" OR "Embryo Transfer" OR "Fertility Preservation")

OR

("Life Style" OR "Lifestyle" OR "Health Behavior" OR "Health Behaviour" OR "Preconception Care") AND ("Female" OR "Women" OR "females" OR "woman" OR "couple" OR "couples") AND ("Fertilization in Vitro" OR "Mitochondrial Replacement Therapy" OR "Intracytoplasmic Sperm Injections" OR "Intracytoplasmic Sperm Injection" OR "Gamete Intrafallopian Transfer" OR "In Vitro Oocyte Maturation" OR "Artificial Insemination")

OR

("Life Style" OR "Lifestyle" OR "Health Behavior" OR "Health Behaviour" OR "Preconception Care") AND ("Female" OR "Women" OR "females" OR "woman" OR "couple" OR "couples") AND ("Oocyte Donation" OR "Oocyte Retrieval" OR "Ovulation Induction" OR "Superovulation" OR "Posthumous Conception" OR "Sperm Retrieval" OR "Zygote Intrafallopian Transfer")

OR

("Life Style" OR "Lifestyle" OR "Health Behavior" OR "Health Behaviour" OR "Preconception Care") AND ("Female" OR "Women" OR "females" OR "woman" OR "couple" OR "couples") AND (("conceive" OR "conception") AND (wish OR wishes OR wishing OR wished))

ClinicalTrials.gov

("Life Style" OR "Lifestyle" OR "Health Behavior" OR "Health Behaviour" OR "Preconception Care") AND ("Female" OR "Women" OR "females" OR "woman" OR "couple" OR "couples")

AND

"infertility" OR "infertile" OR "subfertility" OR "subfertility" OR "preconception period" OR "periconception period" OR "Reproductive Techniques" OR "Assisted Reproduction" OR "Donor Conception" OR "Embryo Transfer" OR "Fertility Preservation"

OR

"Fertilization in Vitro" OR "Mitochondrial Replacement Therapy" OR "Intracytoplasmic Sperm Injections" OR "Intracytoplasmic Sperm Injection" OR "Gamete Intrafallopian Transfer" OR "In Vitro Oocyte Maturation" OR "Artificial Insemination"

OR

"Oocyte Donation" OR "Oocyte Retrieval" OR "Ovulation Induction" OR "Superovulation" OR "Posthumous Conception" OR "Sperm Retrieval" OR "Zygote Intrafallopian Transfer"

OR

(("conceive" OR "conception") AND (wish OR wishes OR wishing OR wished))

S2. List of Excluded Full Text Articles

Citation (Author, Year)	Title	Journal or Trials registry	Reason for Exclusion
Muirhead, R. et al., 2021	A pilot randomized controlled trial of a partial meal replacement preconception weight loss program for women with overweight and obesity	Nutrients	wrong intervention
Agrawal, R et al., 2011	Prospective randomized trial of multiple micronutrients in subfertile women undergoing ovulation induction: a pilot study	Reproductive BioMedicine Online	wrong intervention
den Harink, T et al., 2005	Preconception lifestyle intervention in women with obesity and echocardiographic indices of cardiovascular health in their children	International journal of obesity	wrong population
Den Harink, T et al., 2019	Preconception lifestyle intervention in obese women improves echocardiographic indices of cardiovascular function in their offspring: follow up of a randomised controlled trial	Cardiology in the young	wrong population
Lupiwa, S et al., 1996	Knowledge about sexually transmitted diseases in rural and periurban communities of the Asaro Valley of Eastern Highlands Province: the health education component of an STD study	Papua and New Guinea medical journal	wrong intervention
Maillet, D et al., 2021	Evaluating the Impact of a 6-month Lifestyle Intervention Program on Quality of Life and Motivation in Women With Obesity and Infertility	Canadian journal of diabetes	wrong intervention
Clifton, J et al., 2017	Online Mind/Body Program for Fertility	ClinicalTrials.gov	wrong study design
van der Windt, M et al., 2023	Prototyping of a Digital Life Course Care Platform for Preconception and Pregnancy Care: Pilot Feasibility and Acceptability Study	J Med Internet Res	wrong study design
Mitchell, M et al., 2021	The effect of pre-pregnancy dietary advice and regular exercise to promote weight loss in overweight or obese women on pregnancy outcomes: the BEGIN BETTER randomised trial	WHO ICTRP	not completed
Alibeigi, Z et al., 2020	The Impact of Traditional Medicine-Based Lifestyle and Diet on Infertility Treatment in Women Undergoing Assisted Reproduction: a Randomized Controlled Trial	Complementary medicine research	wrong intervention
Azami, S et al., 2020	Effect of motivational interviewing on dietary intake and weight changes among preconception women with overweight and obesity: a randomized controlled trial	Crescent journal of medical and biological sciences	wrong intervention
Bastani, F et al., 2010	Impact of preconception health education on health locus of control and self-efficacy in women	Eastern Mediterranean health journal	wrong intervention
Belan, M et al., 2019	Lifestyle modifications in male partners of subfertile couples in which the spouse is obese improves the chances of the couple to conceive	Fertility and sterility	wrong intervention
Belan, M et al., 2019	A lifestyle intervention targeting women with obesity and infertility improves their fertility outcomes, especially in women with PCOS: a randomized controlled trial	Fertility and sterility	wrong intervention
Belan, M et al., 2018	Optimizing reproductive health in women with obesity and infertility	Cmaj	wrong study design

Boedt, T et al., 2021	Systematic development of a mobile preconception lifestyle programme for couples undergoing IVF: the PreLiFe-programme	Human reproduction	wrong study design
Cochrane, S et al., 2016	Prior to Conception: the Role of an Acupuncture Protocol in Improving Women's Reproductive Functioning Assessed by a Pilot Pragmatic Randomised Controlled Trial	Evidence-based complementary and alternative medicine	wrong study design
Conceicao, C et al., 2017	Effectiveness of a video intervention on fertility knowledge among university students: a randomised pre-test/post-test study	European Journal of Contraception and Reproductive Health Care	wrong intervention
Kaur, D et al., 2020	Effect of lifestyle modification and education on information needs and satisfaction level of infertile couples	WHO ICTRP	not completed
de Weerd, S et al., 2002	Preconception counseling improves folate status of women planning pregnancy	Obstet Gynecol	wrong intervention
Duval, K et al., 2015	An interdisciplinary lifestyle intervention improves clinically relevant fertility outcomes in obese infertile women-preliminary results of a randomized controlled trial	Canadian journal of diabetes	same study and same outcome
Erkal Aksoy, Y et al., 2019	Women's Health Behaviors Stages of Change (Transtheoretical Model) in Preconception Period: A Randomized Control Study	ClinicalTrials.gov	wrong language
Einarsson, S et al., 2017	Weight reduction intervention for obese infertile women prior to IVF: a randomized controlled trial	Human reproduction	wrong intervention
Einarsson, S et al., 2017	Weight reduction intervention for obese infertile women prior to In vitro fertilisation; a randomised controlled trial	Human reproduction	wrong intervention
Einarsson, S et al., 2018	The effect of weight intervention on obstetric and neonatal outcome in obese women scheduled for IVF treatment	Human reproduction	wrong intervention
Gardiner, P et al., 2020	Using Health Information Technology to Engage African American Women on Nutrition and Supplement Use During the Preconception Period	Frontiers in endocrinology	wrong study design
Harink, T. D et al., 2020	Preconception lifestyle intervention in obese women improves echocardiographic indices of cardiac structure and function in their offspring: follow up of a randomized controlled trial	European journal of preventive cardiology	wrong intervention
Hoirisch-Clapauch, S et al., 2017	Lifestyle modification increases the take-home baby rate in women with recurrent early miscarriages: a randomised study	Thrombosis research	wrong intervention
Hughes, E. G et al., 2000	Randomized trial of a "stage-of-change" oriented smoking cessation intervention in infertile and pregnant women	Fertil Steril	wrong intervention
Salehi, K et al., 2017	The effect of counseling on feeding behavior change among overweight and obese women in pre-pregnancy period	WHO ICTRP	wrong intervention
Ghasemi, S et al., 2016	The effect of preconception counseling on women lifestyle	WHO ICTRP	wrong intervention
Eftkhar, H et al., 2016	the Effect of Self Determination Theory (SDT) based physical activity motivational intervention on happiness and quality of life: a randomized controlled trial	WHO ICTRP	wrong intervention

Eslami, E et al., 2016	The effect of lifestyle based educational package on physical activity and nutritional status in obese and overweight pregnant women	WHO ICTRP	wrong intervention
Ranjbar, F et al., 2020	Effectiveness of an educational intervention on fertility knowledge, childbearing intention and planned pregnancy among new couples	WHO ICTRP	not completed
Kalhori F et al., 2019	Effect of mindfulness-based group counseling on anxiety in infertile women undergoing in vitro fertilization treatment in Hamadan, Iran	Journal of postgraduate medical institute	wrong intervention
Kermack, A. J et al., 2020	Effect of a 6-week "Mediterranean" dietary intervention on in vitro human embryo development: the Preconception Dietary Supplements in Assisted Reproduction double-blinded randomized controlled trial	Fertil Steril	wrong intervention
Koumparou, M et al., 2021	Stress management and In Vitro Fertilization (IVF): A pilot randomized controlled trial	Psychiatriki	wrong intervention
Legro, R. S et al., 2022	Effects of preconception lifestyle intervention in infertile women with obesity: the FIT-PLESE randomized controlled trial	PLoS medicine	wrong intervention
Maas, V. Y. F et al., 2022	The effect of a locally tailored intervention on the uptake of preconception care in the Netherlands: a stepped-wedge cluster randomized trial (APROPOS-II study)	BMC public health	wrong intervention
Maleki, B. H et al., 2017	High-Intensity Exercise Training for Improving Reproductive Function in Infertile Patients: a Randomized Controlled Trial	Journal d'obstetrique et gynecologie du Canada	wrong intervention
Mintjens, S et al., 2021	The Effects of a Preconception Lifestyle Intervention on Childhood Cardiometabolic Health-Follow-Up of a Randomized Controlled Trial	Cells	wrong intervention
Mori, A., 2009	Supporting stress management for women undergoing the early stages of fertility treatment: a cluster-randomized controlled trial	Jpn J Nurs Sci	wrong intervention
Mutsaerts, M. A., 2016	Randomized trial of a lifestyle program in obese infertile women	Nederlands tijdschrift voor geneeskunde	wrong intervention
Rothberg, A. E et al., 2013	The Effects of an Intensive Lifestyle Intervention on Reproductive Outcomes	ClinicalTrials.gov	wrong intervention
Santoro, N et al., 2015	Improving Reproductive Fitness Through Pretreatment With Lifestyle Modification in Obese Women With Unexplained Infertility	ClinicalTrials.gov	wrong intervention
Lie Fong, S et al., 2018	Evaluation of a Mobile Preconception Lifestyle Programme in Couples Undergoing In Vitro Fertilisation	ClinicalTrials.gov	wrong study design
Lisón Párraga, J. F. et al., 2020	Internet-based Intervention to Promote a Healthy Lifestyle on the Reproductive Parameters of Overweight and Obese Women	ClinicalTrials.gov	not completed
Fraser, W. D. et al., 2020	Healthy for my Baby- RCT of a Lifestyle Intervention for Overweight Women in Preconception	ClinicalTrials.gov	not completed
Erasmus Medical Centre, 2019	APROPOS-II Study	WHO ICTRP	not completed
Norris, S. A et al., 2022	Building knowledge, optimising physical and mental health and setting up healthier life trajectories in South African women (Bukhali): a preconception randomised control trial part of the Healthy Life Trajectories Initiative (HeLTI)	BMJ open	wrong intervention

Oostingh, E. C et al., 2020	Improvement of periconception nutrition and lifestyle behaviors using the mhealth program smarter pregnancy: a randomized controlled trial	Reproductive sciences	wrong study design
Ozturk, B et al., 2020	Effects of infertility prevention programme on college students	Sex Education	wrong intervention
Sant'Anna, E. M et al., 2017	Mindfulness-based intervention for lifestyle modification and weight loss in infertile women: randomized controlled trial	Human reproduction	wrong intervention
Sant'Anna, E. M et al., 2022	Mindfulness-based program to support lifestyle modification and weight loss in infertile women: randomized controlled trial	Journal of psychosomatic obstetrics and gynaecology	wrong intervention
Santos, C et al., 2017	Effects of life events on infertility diagnosis: comparison with presumably fertile men and women	J Reprod Infant Psychol	wrong intervention
Stern, J et al., 2013	Introducing reproductive life plan-based information in contraceptive counselling: an RCT	Human reproduction	wrong intervention
Tolahunase, M et al., 2018	Impact of yoga- and meditation based lifestyle intervention on depression and quality of life in infertile couples: a randomized controlled trial	Human reproduction. Conference:34th annual meeting of the european society of human reproduction and embryology.	wrong intervention
Tolahunase, M. R et al., 2018	Impact of yoga- and meditation-based lifestyle intervention on depression, quality of life, and cellular aging in infertile couples	Fertility and sterility. Conference: 74th annual congress of the american society for reproductive medicine	same study and same outcome
Tuil, W. S. et al., 2007	Empowering patients undergoing in vitro fertilization by providing Internet access to medical data	Fertil Steril	wrong intervention
van Dammen, L et al., 2021	A lifestyle intervention randomized controlled trial in obese women with infertility improved body composition among those who experienced childhood adversity	Stress and health	wrong intervention
van Dammen, L et al., 2020	A lifestyle intervention RCT in obese women with infertility improved body composition among those who experienced childhood adversity	Stress and health	wrong intervention
van Dijk, M. R et al., 2020	A Mobile App Lifestyle Intervention to Improve Healthy Nutrition in Women Before and During Early Pregnancy: single-Center Randomized Controlled Trial	Journal of medical Internet research	wrong population
van Dijk, M. R et al., 2017	The use of the mHealth program Smarter Pregnancy in preconception care: rationale, study design and data collection of a randomized controlled trial	BMC pregnancy and childbirth	wrong study design
van Elten, T. M et al., 2019	Preconception lifestyle intervention reduces long term energy intake in women with obesity and infertility: a randomised controlled trial	International journal of behavioral nutrition and physical activity	wrong study design
van Elten, T. M et al., 2018	Effects of a preconception lifestyle intervention in obese infertile women on diet and physical activity; A secondary analysis of a randomized controlled trial	PloS one	wrong intervention

van Elten, T. M et al., 2019	Preconception Lifestyle and Cardiovascular Health in the Offspring of Overweight and Obese Women	Nutrients	wrong intervention
Van Oers, A et al., 2016	Is there a different effect of lifestyle intervention in subgroups of infertile obese women? Prespecified subgroup analyses of the LIFEstyle randomised controlled trial	Human reproduction	wrong intervention
van Oers, A. M et al., 2016	Effectiveness of lifestyle intervention in subgroups of obese infertile women: a subgroup analysis of a RCT	Human reproduction	wrong intervention
Vause, T. D. R et al., 2018	Comparison of a Web-Based Teaching Tool and Traditional Didactic Learning for In Vitro Fertilization Patients: A Preliminary Randomized Controlled Trial	J Obstet Gynaecol Can	wrong intervention
Wang, J. X et al., 2018	Positive Effect of Acupuncture and Cupping in Infertility Treatment	Medical Acupuncture	wrong study design
Wang, Z. et al., 2021	The effect of lifestyle intervention on systemic oxidative stress in women with obesity and infertility: a post-hoc analysis of a randomized controlled trial	Journal of clinical medicine	wrong intervention
Wang, Z.et al., 2021	Effectiveness of a 6-month lifestyle intervention on diet, physical activity, quality of life, and markers of cardiometabolic health in women with pcos and obesity and non-pcos obese controls: one size fits all?	Nutrients	wrong intervention
Wekker, V. et al., 2018	A lifestyle intervention improves sexual function of women with obesity and infertility: a 5 year follow-up of a RCT	PloS one	wrong intervention
Wekker, V. et al., 2017	Effect of a lifestyle intervention in obese infertile women on cardiometabolic health and quality of life: results of a randomised controlled trial	Human reproduction	wrong intervention
Yokomizo, R et al., 2021	Smartphone application improves fertility treatmentrelated literacy: a large-scale surveillance and randomized controlled trial in Japan	Human reproduction	wrong intervention
